# Supplementary material for: Direct and indirect effects of land-use intensity on plant communities across elevation in semi-natural grasslands
Source: PLoS One. 2020 Nov 24;15(11):e0231122. doi: 10.1371/journal.pone.0231122 (PMC7685434; doi:10.1371/journal.pone.0231122)
Supplement: S1 Appendix — (DOCX) [file pone.0231122.s001.docx]

Supporting Information for

**Direct and indirect effects of land-use intensity on plant communities**

**across elevation in semi-natural grasslands**

Oksana Y. Buzhdygan [
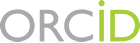
](https://orcid.org/0000-0003-1226-7864), Britta Tietjen [
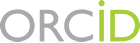
](https://orcid.org/0000-0003-4767-6406), Svitlana S. Rudenko, Volodymyr A. Nikorych[
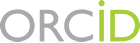
](https://orcid.org/0000-0001-5006-4027), Jana S. Petermann [
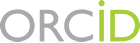
](https://orcid.org/0000-0002-3898-5656).

^*^Correspondence to: [oksana.buzh@fu-berlin.de](mailto:oksana.buzh@fu-berlin.de),
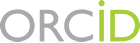
 https://orcid.org/0000-0003-1226-7864;

[jana.petermann@sbg.ac.at](mailto:jana.petermann@sbg.ac.at),
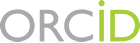
 https://orcid.org/0000-0002-3898-5656.


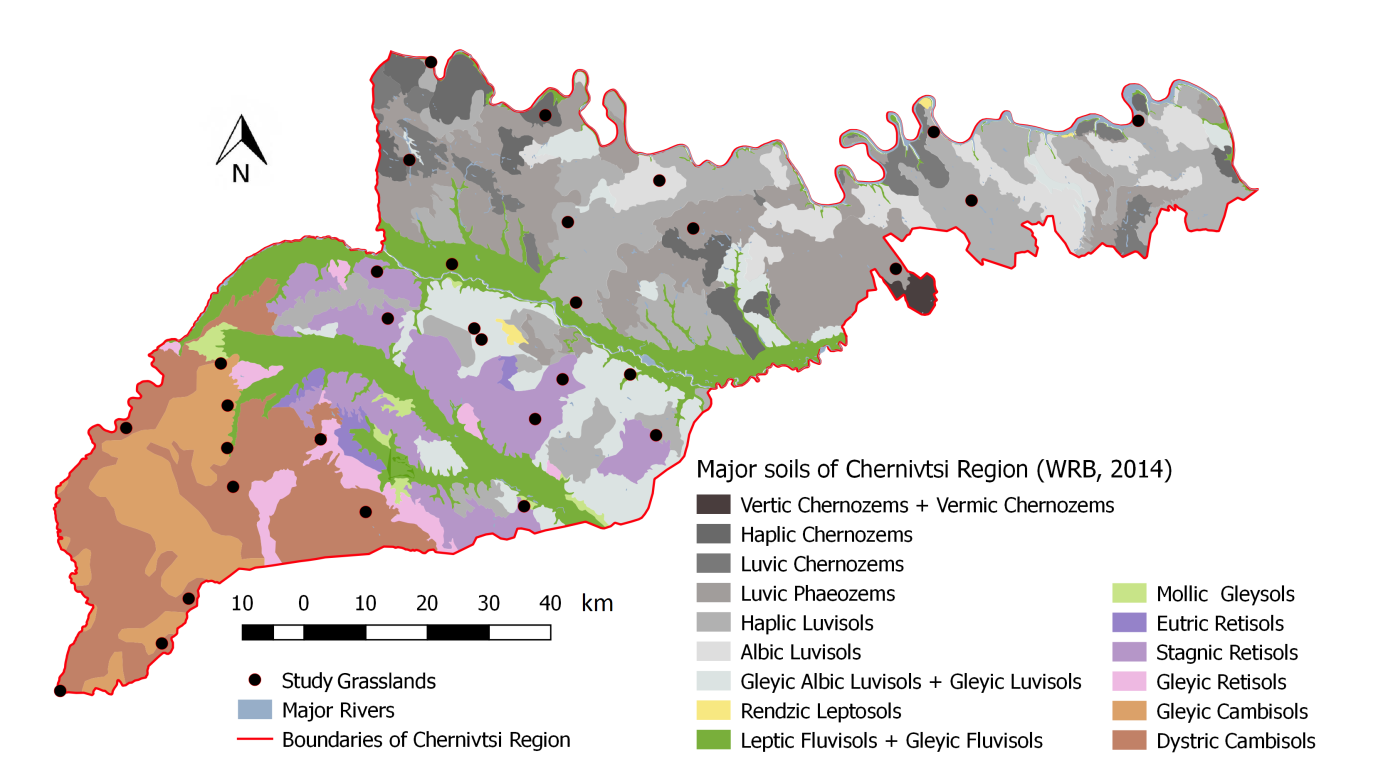


**S1 Fig. Soil map.** Soil types of the study area (Chernivtsi Region, Ukraine) and study grassland sites (see also Fig. 2, main text). Soil types are in accordance with the international soil classification system (World reference base for soil resources, WRB 2014 [1]). Soil map was adapted from Polchyna et al. [2] with permission. Map was created using QGIS 2.18.26 [3].

**S2 Fig. Original SEM model.** SEM model testing the interactive effects of grazing and elevation (purple dashed paths) on plant diversity and composition. Numbers associated with each arrow are standardized path coefficients with the following levels of significance: *P ≤ 0.05; **P ≤ 0.01; ***P ≤ 0.001. Purple dashed paths show the interactive effects (not significant) of grazing and elevation (Cattle density : Elevation). Red and black paths represent significant (i.e., P ≤ 0.05) negative and positive path coefficients, respectively, and grey paths were not significant. Model fit statistics: χ2 = 62.3, df = 15, P = 0; RMSEA = 0.3, PRMSEA = 0; CFI = 0.76; NNFI = 0.14; SRMR = 0.14, where RMSEA: Root Mean Square Error of Approximation; CFI, Comparative Fit Index; NNFI: Tucker–Lewis Non-Normed Fit Index; SRMR: Standardized Root Mean Square Residual. The not significant interactive effects of grazing and elevation on plant species richness, functional diversity, and proportion of undesirable species were removed in the final model (Fig. 3, main text, S6 Table) to acquire the adequate fit statistics.

**S1 Table. Hypothesized mechanisms.** Hypothesized relationships between the variables in Fig. 1, based on the literature.

| **Path №** (Fig. 1) | **Path** | **Direction of effects** | **Hypothesized mechanisms** |
| --- | --- | --- | --- |
| **1** | **Cattle density**  **🡪**  **Plant diversity and composition** | negative  negative  negative  negative  positive  negative  positive | ***Physical damage***:  - Heavy livestock directly physically destroys vegetation by trampling [4];  - Cattle manure blocks plant growth [5].  ***Chemical damage***:  Cattle urine burns plants and it is toxic to roots [5].  ***Biological impact***:  Cattle spread plant pathogen infections [6].  ***Altered local colonization processes***:  - Grazing removes seeds and reproductive structures of plants, therefore decreasing availability of propagules of extant species [7,8];  - Cattle import the propagules of new species to grassland via seed dispersal [8]. This can result in increase in abundance of invasive plant species.  ***Altered competition processes***:  Direct consumption of competitively dominant plants species [4,8] can relax competitive interactions among plant species [9]. |
| **2** | **Cattle density**  **🡪**  **Bare soil exposure** | positive | Heavy livestock compacts soil structure, resulting in loss of vegetation cover and in bare soil exposure [10]. |
| **3** | **Bare soil exposure**  **🡪**  **Plant diversity and composition** | negative  negative  negative  positive | Bare soil as a result of soil compaction:  - prevents the restoration of vegetation [11,12];  - reduces the use efficiency of available nutrients by plants [13,14].  The vegetation gaps enhance soil evaporation and plant evapotranspiration [8].  ***Altered local colonization processes***:  Moderate level of soil disturbances may stimulate germination of plants from the soil seed bank as a result of increased light availability and nutrient-rich and pathogen-free soil in vegetation gaps [8,15]. |
| **4** | **Cattle density**  **🡪**  **Earthworms**  **and soil microorganisms** | positive  positive  negative  negative | Deposition of excrements by cattle into soil serves as the resource input [16] for earthworms and soil microorganisms [17,18].  Cattle manure is partly microbial in composition derived from fermentation during digestion [5] Input of faecal bacteria into soil through the deposition of cattle manures may increase soil bacterial biodiversity and abundance [19].  Cattle urine increases microbial stress and alters microbial community structure [20].  Cattle manure and urine depositions may contain veterinary treatment additives [5], which can be toxic to earthworms [21] and soil microorganisms [22] and can reduce soil biodiversity [23]. |
| **5** | **Earthworms**  **and soil microorganisms**  **🡪**  **Plant diversity and composition** | positive  positive / negative | Earthworms and soil microorganisms impact nutrient cycling and resource availability for plants [24,25].  Decomposition, burrowing, casting and mixing activities of earthworms are found to affect growth [24,25] and competitive interactions among grassland plant species [26,27]. |
| **6** | **Bare soil exposure**  **🡪**  **Earthworms**  **and soil microorganisms** | negative  negative  positive | Bare soil as a result of soil compaction:  - reduces earthworm density, biomass [24], and burrowing and casting activity [28,29];  - decreases soil microbial biomass [30,31] and inhibits microbial activity [32];  - reduces soil porosity [24] which physically protects microorganisms from predating protozoa and nematodes [24,31]. |
| **7** | **Earthworms**  **and soil microorganisms** **🡪**  **Soil Organic Carbon (SOC)** | positive | Earthworms and soil microorganisms play a major role in the decomposition processes in temperate grasslands [33–36]. |
| **8** | **Soil properties**  **(SOC, pH)**  **🡪**  **Plant diversity and composition** | positive  positive  positive | Soil organic matter serves as a resource to plants [16].  Increasing soil pH (up to nearly neutral or slightly alkaline values) increases the nutrient uptake by plants.  Low pH (i.e., acidic) soils expose plants to aluminium toxicity [37]. |
| **9** | **Cattle density**  **🡪**  **Soil properties**  **(SOC, pH)** | positive  positive | Cattle manure deposition increases soil organic matter [38].  Cattle manure and urine relax soil acidification [20,39] and maintain soil pH in the optimal range for plants [40]. |
| **10** | **Bare soil exposure**  **🡪**  **Soil properties**  **(SOC, pH)** | negative | Bare soil as a result of soil compaction reduces soil porosity [24] which in turn slows down decomposition of organic matter [32,41]. |
| **11** | **Elevation**  **🡪**  **Plant diversity and composition** | positive / negative  positive | ***Environmental filtering***:  Altered climatic conditions along the elevation gradient shape regional species pool composition [42].  ***Habitat diversity***:  Higher topographic variability and mosaic bedrock, nutrient availability, and humidity linked to steep elevation gradients in the Carpathian Mountains and their foothills result in greater habitat diversity [43], and therefore may lead to increased plant species richness. |
| **12** | **Elevation**  **🡪**  **Cattle density** | negative | Lower grazing intensity is expected with increasing elevation [44]. |
| **13** | **Elevation**  **🡪**  **Soil properties**  **(SOC, pH)** | positive / negative  negative | Variations in the soil types across the different physical-geographical zones (i.e., Luvisols and Chernozems predominate in the plains, acidic Retisols in the foothills, and Cambisols in the mountains, Fig. S1) shape changes in the soil properties along the elevation gradient [2,45].  Higher soil leaching processes in humid and low temperature conditions with increasing elevation is expected to increase soil acidity [46]. |
| **14** | **Soil pH**  **🡪**  **Earthworms**  **and soil microorganisms** | positive | With increasing pH (up to pH nearly neutral or slightly alkaline) the number and activity of earthworms and of bacteria increases [35]. |
| **15** | **Elevation**  **🡪**  **Bare soil exposure** | negative | With increasing elevation extensive natural erosion processes with heavy runoff and soil losses are expected to have more pronounced effects on bare soil exposure [47]. |
| **An interactive effect:** | | | |
| **16** | **Cattle density : Elevation**  **🡪**  **Plant diversity and composition** | positive / negative | Similar levels of grazing intensity in the mountains might show different (i.e., stronger or weaker) effects than those in plains. |

**S2 Table. Conversion factors used to transform the number of cattle to livestock units based on the type and age of animals.** The conversion factors are widely used coefficients in Europe established initially on the basis of the nutritional or feed requirement of each type of animal [48].

**B**

| **Type and age of**  **cattle animals** | **Livestock** **unit** |
| --- | --- |
| dairy cows | 1.0 |
| males, 2 years old and over | 1.0 |
| other cows, 2 years old and over | 0.8 |
| female cows that have not yet calved (heifers), 2 years old and over | 0.8 |
| animals from 1 to 2 years old | 0.7 |
| animals under 1 year old | 0.4 |

**S3 Table. Summary statistics for plant community variables.** Sum, minimum (min), maximum (max) and mean values, and standard errors ±S.E. of the richness are derived from the plant community data for each of the physical-geographical zones and across the entire region; n is the number of study grasslands.

| **Study area** | **Summary statistics** | **Total plant community** | **Functional group:** | | | | **Functional diversity** | **Undesirable species** |
| --- | --- | --- | --- | --- | --- | --- | --- | --- |
|  |  |  | Legumes | Grasses | Rushes and sedges | Forbs other than legumes |  |  |
| Across  the entire region (n=31) | sum | 175 | 13 | 18 | 8 | 136 | – – | 86 |
|  | min | 7 | 0 | 0 | 0 | 5 | 1 | 5 |
|  | max | 50.5 | 6 | 10 | 3 | 41.5 | 4 | 28.5 |
|  | mean | 27.5 | 2.95 | 2.71 | 0.55 | 21.3 | 3.13 | 13.87 |
|  | ±S.E. | 1.97 | 0.27 | 0.42 | 0.15 | 1.54 | 0.15 | 1.08 |
| Physical-geographical zone: | | | | | | | | |
| Plains  (n=12) | sum | 101 | 7 | 10 | 6 | 78 | – – | 48 |
|  | min | 7 | 0 | 0 | 0 | 5 | 1 | 5 |
|  | max | 36.5 | 4 | 5 | 2 | 28.5 | 4 | 19 |
|  | mean | 20.1 | 2.25 | 1.92 | 0.42 | 15.5 | 2.92 | 10.13 |
|  | ± S.E. | 2.38 | 0.35 | 0.54 | 0.19 | 1.79 | 0.31 | 1.16 |
| Foothills  (n=12) | sum | 123 | 11 | 9 | 3 | 100 | – – | 60 |
|  | min | 15.5 | 1 | 0 | 0 | 12.5 | 2 | 8.5 |
|  | max | 50.5 | 6 | 6 | 2 | 41.5 | 4 | 28.5 |
|  | mean | 29.8 | 3.38 | 2.92 | 0.33 | 23.13 | 3.17 | 15.17 |
|  | ± S.E. | 2.84 | 0.50 | 0.48 | 0.19 | 2.35 | 0.17 | 1.68 |
| Mountains  (n=7) | sum | 116 | 7 | 13 | 7 | 89 | – – | 62 |
|  | min | 21 | 2 | 0 | 0 | 15 | 2 | 9 |
|  | max | 44.5 | 5 | 10 | 3 | 34.5 | 4 | 27 |
|  | mean | 36.5 | 3.43 | 3.71 | 1.14 | 28.29 | 3.43 | 18.07 |
|  | ± S.E. | 3.47 | 0.43 | 1.39 | 0.40 | 2.56 | 0.30 | 2.33 |

**S4 Table. List of plant species sampled across all study grasslands (n=31) and their classification into functional groups and as undesirable species.**

| **Plant species**  (sorted by family) | **Family** | **Plant traits:** | | | | |
| --- | --- | --- | --- | --- | --- | --- |
|  |  | **Functional group** | | | | **Undesirable species**  [49–51] |
|  |  | Legumes | Grasses | Rushes and sedges | Forbs other than legumes |  |
| Anthyllis marcocephala Wend. | Fabaceae | 1 | 0 | 0 | 0 | 0 |
| Coronilla varia L. | Fabaceae | 1 | 0 | 0 | 0 | 1 |
| Lathyrus sylvestris L. | Fabaceae | 1 | 0 | 0 | 0 | 0 |
| Medicago lupulina L. | Fabaceae | 1 | 0 | 0 | 0 | 0 |
| Melilotus officinalis (L.) Pall. | Fabaceae | 1 | 0 | 0 | 0 | 0 |
| Trifolium arvense L. | Fabaceae | 1 | 0 | 0 | 0 | 0 |
| Trifolium campestre Schreb. | Fabaceae | 1 | 0 | 0 | 0 | 0 |
| Trifolium pannonicum Jacq. | Fabaceae | 1 | 0 | 0 | 0 | 0 |
| Trifolium pratense L. | Fabaceae | 1 | 0 | 0 | 0 | 0 |
| Trifolium repens L | Fabaceae | 1 | 0 | 0 | 0 | 0 |
| Trifolium sativum (Schreb.) Crome | Fabaceae | 1 | 0 | 0 | 0 | 0 |
| Vicia cracca L. | Fabaceae | 1 | 0 | 0 | 0 | 0 |
| Vicia sativa L. | Fabaceae | 1 | 0 | 0 | 0 | 0 |
| Agrostis stolonifera L. | Poaceae | 0 | 1 | 0 | 0 | 0 |
| Agrostis tenuis Sibth. | Poaceae | 0 | 1 | 0 | 0 | 0 |
| Alopecurus pratensis L. | Poaceae | 0 | 1 | 0 | 0 | 0 |
| Briza media L. | Poaceae | 0 | 1 | 0 | 0 | 0 |
| Calamagrostis arundinacea L. | Poaceae | 0 | 1 | 0 | 0 | 0 |
| Cynosurus cristatus L. | Poaceae | 0 | 1 | 0 | 0 | 0 |
| Dactylis glomerata L. | Poaceae | 0 | 1 | 0 | 0 | 0 |
| Elytrigia repens (L.) Nevski | Poaceae | 0 | 1 | 0 | 0 | 0 |
| Festuca pratensis Heuff. | Poaceae | 0 | 1 | 0 | 0 | 0 |
| Festuca rubra L. | Poaceae | 0 | 1 | 0 | 0 | 0 |
| Koeleria cristata (L.) Pers. | Poaceae | 0 | 1 | 0 | 0 | 0 |
| Melica nutans L. | Poaceae | 0 | 1 | 0 | 0 | 0 |
| Phalaroides arundinaceae (L.) Rausch. | Poaceae | 0 | 1 | 0 | 0 | 0 |
| Phleum pratense L. | Poaceae | 0 | 1 | 0 | 0 | 0 |
| Phragmites australis (Cav.) Trin. Ex Steud. | Poaceae | 0 | 1 | 0 | 0 | 0 |
| Poa trivialis L. | Poaceae | 0 | 1 | 0 | 0 | 0 |
| Bromus arvensis L. | Poaceae | 0 | 1 | 0 | 0 | 0 |
| Avena fatua L. | Poaceae | 0 | 1 | 0 | 0 | 0 |
| Allium victorialis L. | Alliaceae | 0 | 0 | 0 | 1 | 1 |
| Majanthemum bifolium (L.) F. W. Schmidt | Alliaceae | 0 | 0 | 0 | 1 | 1 |
| Pimpinella saxifraga L. | Apiaceae | 0 | 0 | 0 | 1 | 0 |
| Eryngium campestre L. | Apiaceae | 0 | 0 | 0 | 1 | 1 |
| Carum carvi L. | Apiaceae | 0 | 0 | 0 | 1 | 0 |
| Aegopodium podagraria L. | Apiaceae | 0 | 0 | 0 | 1 | 0 |
| Dryopteris filix-mas (L.) Schott | Aspidiaceae | 0 | 0 | 0 | 1 | 1 |
| Achillea millefolium Klok. et Krytzka | Asteraceae | 0 | 0 | 0 | 1 | 1 |
| Anthemis arvensis L. | Asteraceae | 0 | 0 | 0 | 1 | 1 |
| Artemisia absinthium L. | Asteraceae | 0 | 0 | 0 | 1 | 1 |
| Bellis perennis L. | Asteraceae | 0 | 0 | 0 | 1 | 1 |
| Carduus crispus L. | Asteraceae | 0 | 0 | 0 | 1 | 1 |
| Carlina acaulis L. | Asteraceae | 0 | 0 | 0 | 1 | 1 |
| Centaurea cyanus L. | Asteraceae | 0 | 0 | 0 | 1 | 1 |
| Centaurea jacea L. | Asteraceae | 0 | 0 | 0 | 1 | 1 |
| Сentaurea stricta Waldst. et Kit. | Asteraceae | 0 | 0 | 0 | 1 | 1 |
| Chamomilla recutita (L.) Rauschert | Asteraceae | 0 | 0 | 0 | 1 | 0 |
| Cichorium intybus L. | Asteraceae | 0 | 0 | 0 | 1 | 0 |
| Cirsium arvense (L.) Scop. | Asteraceae | 0 | 0 | 0 | 1 | 1 |
| Cirsium vulgare (Savi) Ten. | Asteraceae | 0 | 0 | 0 | 1 | 1 |
| Crepis tectorum L. | Asteraceae | 0 | 0 | 0 | 1 | 1 |
| Galinsoga parviflora Cav. | Asteraceae | 0 | 0 | 0 | 1 | 0 |
| Hieracium villosum Jacq. | Asteraceae | 0 | 0 | 0 | 1 | 1 |
| Inula salicina L. | Asteraceae | 0 | 0 | 0 | 1 | 0 |
| Lapsana comnunis L. | Asteraceae | 0 | 0 | 0 | 1 | 1 |
| Leontodon hispidus L. | Asteraceae | 0 | 0 | 0 | 1 | 0 |
| Leucanthemum vulgare Lam. | Asteraceae | 0 | 0 | 0 | 1 | 0 |
| Matricaria perforata Merat | Asteraceae | 0 | 0 | 0 | 1 | 1 |
| Paludosa (L.) Moench | Asteraceae | 0 | 0 | 0 | 1 | 0 |
| Pyrethrum corymbosum (L.) Scop. | Asteraceae | 0 | 0 | 0 | 1 | 0 |
| Stenactis annua Nees. | Asteraceae | 0 | 0 | 0 | 1 | 1 |
| Tanacetum millefolium (L.) Tzvel. | Asteraceae | 0 | 0 | 0 | 1 | 1 |
| Taraxacum officinale Webb. ex Wigg. | Asteraceae | 0 | 0 | 0 | 1 | 0 |
| Tragopogon pratensis L. | Asteraceae | 0 | 0 | 0 | 1 | 0 |
| Tussilago farfara L. | Asteraceae | 0 | 0 | 0 | 1 | 0 |
| Borago officinalis L. | Boraginaceae | 0 | 0 | 0 | 1 | 1 |
| Cynoglossum officinale L. | Boraginaceae | 0 | 0 | 0 | 1 | 1 |
| Echium vulgare L. | Boraginaceae | 0 | 0 | 0 | 1 | 1 |
| Myosotis arvensis L. | Boraginaceae | 0 | 0 | 0 | 1 | 0 |
| Symphytum cordatum Waldst. Et Kit. Ex Willd. | Boraginaceae | 0 | 0 | 0 | 1 | 0 |
| Barbarea vulgaris R. Br. | Brassicaceae | 0 | 0 | 0 | 1 | 0 |
| Berteroa incana (L.) DC. | Brassicaceae | 0 | 0 | 0 | 1 | 1 |
| Capsella bursa-pastoris (L.) Medic | Brassicaceae | 0 | 0 | 0 | 1 | 0 |
| Cardamine pratensis L. | Brassicaceae | 0 | 0 | 0 | 1 | 1 |
| Lepidium ruderale L. | Brassicaceae | 0 | 0 | 0 | 1 | 0 |
| Lunaria rediviva L. | Brassicaceae | 0 | 0 | 0 | 1 | 1 |
| Rorippa palustris (L.) Bess. | Brassicaceae | 0 | 0 | 0 | 1 | 1 |
| Thlaspi arvense L. | Brassicaceae | 0 | 0 | 0 | 1 | 0 |
| Sambucus nigra L. | Caprifoliaceae | 0 | 0 | 0 | 1 | 1 |
| Scabiosa ochroleuca L. | Caprifoliaceae | 0 | 0 | 0 | 1 | 0 |
| Scabiosa opaca Klok. | Caprifoliaceae | 0 | 0 | 0 | 1 | 0 |
| Coronaria flos-cuculi (L.) A. Br. | Caryophyllalceae | 0 | 0 | 0 | 1 | 0 |
| Dianthus campestris Bieb. | Caryophyllalceae | 0 | 0 | 0 | 1 | 0 |
| Dianthus compactus Kit. | Caryophyllalceae | 0 | 0 | 0 | 1 | 0 |
| Dianthus deltoides L. | Caryophyllalceae | 0 | 0 | 0 | 1 | 0 |
| Melandrium album Garche. | Caryophyllalceae | 0 | 0 | 0 | 1 | 0 |
| Saponaria officinalis L. | Caryophyllalceae | 0 | 0 | 0 | 1 | 1 |
| Silene nutans L. | Caryophyllalceae | 0 | 0 | 0 | 1 | 1 |
| Stellaria graminea L. | Caryophyllalceae | 0 | 0 | 0 | 1 | 1 |
| Trollius europaeus L. | Caryophyllalceae | 0 | 0 | 0 | 1 | 1 |
| Chenopodium album L. | Chenopodiaceae | 0 | 0 | 0 | 1 | 0 |
| Chenopodium hybridum L. | Chenopodiaceae | 0 | 0 | 0 | 1 | 0 |
| Convolvulus arvensis L. | Convolvulaceae | 0 | 0 | 0 | 1 | 1 |
| Carex hordeistichos Vill. | Cyperaceae | 0 | 0 | 1 | 0 | 1 |
| Carex leporina L. | Cyperaceae | 0 | 0 | 1 | 0 | 1 |
| Carex sylvatica Huds. | Cyperaceae | 0 | 0 | 1 | 0 | 1 |
| Eriophorum polystachyon L. | Cyperaceae | 0 | 0 | 1 | 0 | 1 |
| Nardus stricta L. | Cyperaceae | 0 | 0 | 1 | 0 | 1 |
| Equisetum arvense L. | Equisetaceae | 0 | 0 | 0 | 1 | 1 |
| Euphorbia cyparissias L. | Euphorbiaceae | 0 | 0 | 0 | 1 | 1 |
| Euphorbia peplus L. | Euphorbiaceae | 0 | 0 | 0 | 1 | 1 |
| Centaurium erythraea Rafn | Gentianaceae | 0 | 0 | 0 | 1 | 1 |
| Erodium cicutarium (L.) L’Her. | Geraniaceae | 0 | 0 | 0 | 1 | 1 |
| Linum usitatissimum L. | Geraniaceae | 0 | 0 | 0 | 1 | 0 |
| Hypericum perforatum L. | Hypericaceae | 0 | 0 | 0 | 1 | 1 |
| Gladiolus imbricatus L. | Iridaceae | 0 | 0 | 0 | 1 | 1 |
| Juncus bufonius L. | Juncaceae | 0 | 0 | 1 | 0 | 1 |
| Luzula pilosa (L.) Willd. | Juncaceae | 0 | 0 | 1 | 0 | 1 |
| Ajuga reptans L. | Lamiaceae | 0 | 0 | 0 | 1 | 0 |
| Betonica officinalis L. s. l. | Lamiaceae | 0 | 0 | 0 | 1 | 1 |
| Glechoma hederacea L. | Lamiaceae | 0 | 0 | 0 | 1 | 1 |
| Lamium album L. | Lamiaceae | 0 | 0 | 0 | 1 | 1 |
| Mentha arvensis L. | Lamiaceae | 0 | 0 | 0 | 1 | 1 |
| Origanum vulgare L. | Lamiaceae | 0 | 0 | 0 | 1 | 1 |
| Prunella vulgaris L. | Lamiaceae | 0 | 0 | 0 | 1 | 0 |
| Salvia nemorosa L. | Lamiaceae | 0 | 0 | 0 | 1 | 1 |
| Salvia verticillata L. | Lamiaceae | 0 | 0 | 0 | 1 | 1 |
| Stachys germanica L. | Lamiaceae | 0 | 0 | 0 | 1 | 1 |
| Stachys palustris L. | Lamiaceae | 0 | 0 | 0 | 1 | 1 |
| Thumus serpyllum L. | Lamiaceae | 0 | 0 | 0 | 1 | 1 |
| Lythrum salicaria L. | Lythraceae | 0 | 0 | 0 | 1 | 1 |
| Malva mauritiana L. | Malvaceae | 0 | 0 | 0 | 1 | 0 |
| Gymnadenia conopsea (L.) R. Br. | Orchidaceae | 0 | 0 | 0 | 1 | 0 |
| Platanthera bifolia (L.) Rich. | Orchidaceae | 0 | 0 | 0 | 1 | 0 |
| Oxalis acetosella L. | Oxalidaceae | 0 | 0 | 0 | 1 | 1 |
| Chelidonium majus L. | Papaveraceae | 0 | 0 | 0 | 1 | 1 |
| Papaver rhoeas L. | Papaveraceae | 0 | 0 | 0 | 1 | 1 |
| Plantago lanceolata L. | Plantaginaceae | 0 | 0 | 0 | 1 | 1 |
| Plantago major L. | Plantaginaceae | 0 | 0 | 0 | 1 | 1 |
| Plantago media L. | Plantaginaceae | 0 | 0 | 0 | 1 | 1 |
| Polygala vulgaris L. | Polygalales | 0 | 0 | 0 | 1 | 1 |
| Polygonum aviculare L. | Polygonaceae | 0 | 0 | 0 | 1 | 0 |
| Polygonum bistorta L. | Polygonaceae | 0 | 0 | 0 | 1 | 0 |
| Polygonum hydropiper L. | Polygonaceae | 0 | 0 | 0 | 1 | 1 |
| Rumex confertus Willd. | Polygonaceae | 0 | 0 | 0 | 1 | 0 |
| Rumex crispus L. | Polygonaceae | 0 | 0 | 0 | 1 | 0 |
| Anagallis arvensis L. | Primulaceae | 0 | 0 | 0 | 1 | 1 |
| Lysimachia nummularia L. | Primulaceae | 0 | 0 | 0 | 1 | 1 |
| Primula vulgaris Huds. | Primulaceae | 0 | 0 | 0 | 1 | 0 |
| Consolida regalis S. F. Gray | Ranunculaceae | 0 | 0 | 0 | 1 | 1 |
| Ranunculus acris L | Ranunculaceae | 0 | 0 | 0 | 1 | 1 |
| Ranunculus repens L. | Ranunculaceae | 0 | 0 | 0 | 1 | 1 |
| Agrimonia eupatoria L. | Rosaceae | 0 | 0 | 0 | 1 | 0 |
| Agrimonia grandis Andrz. ex. C. A. Mey. | Rosaceae | 0 | 0 | 0 | 1 | 0 |
| Alchemilla alpestris F.W. Schmidt | Rosaceae | 0 | 0 | 0 | 1 | 0 |
| Aruncus vulgaris Rafin | Rosaceae | 0 | 0 | 0 | 1 | 1 |
| Crataegus ucrainica Pojark | Rosaceae | 0 | 0 | 0 | 1 | 1 |
| Filipendula denudata (J et. C. Presl) Fritsch. | Rosaceae | 0 | 0 | 0 | 1 | 0 |
| Filipendula vulgaris Moench. | Rosaceae | 0 | 0 | 0 | 1 | 0 |
| Fragaria vesca L. | Rosaceae | 0 | 0 | 0 | 1 | 0 |
| Geum urbanum L. | Rosaceae | 0 | 0 | 0 | 1 | 0 |
| Lotus arvensis Pers. | Rosaceae | 0 | 0 | 0 | 1 | 0 |
| Potentilla argentea L. | Rosaceae | 0 | 0 | 0 | 1 | 0 |
| Potentilla anserina L. | Rosaceae | 0 | 0 | 0 | 1 | 1 |
| Potentilla erecta (L.) Raeusch. | Rosaceae | 0 | 0 | 0 | 1 | 0 |
| Potentilla reptans L. | Rosaceae | 0 | 0 | 0 | 1 | 0 |
| Rosa canina L. | Rosaceae | 0 | 0 | 0 | 1 | 0 |
| Galium verum L. | Rubiaceae | 0 | 0 | 0 | 1 | 0 |
| Asperula graveolens Bieb. ex Schult. et Schult. fil. | Rubiaceae | 0 | 0 | 0 | 1 | 0 |
| Galium aparine L. | Rubiaceae | 0 | 0 | 0 | 1 | 1 |
| Euphrasia montana Jord. | Scrophulariaceae | 0 | 0 | 0 | 1 | 1 |
| Melampyrum nemorosum L. | Scrophulariaceae | 0 | 0 | 0 | 1 | 0 |
| Rhinanthus minor L. | Scrophulariaceae | 0 | 0 | 0 | 1 | 1 |
| Verbascum thapsus L. | Scrophulariaceae | 0 | 0 | 0 | 1 | 1 |
| Veronica longifolia L. | Scrophulariaceae | 0 | 0 | 0 | 1 | 1 |
| Veronica urticifolia Jacq. | Scrophulariaceae | 0 | 0 | 0 | 1 | 0 |
| Urtica urens L. | Urticaceae | 0 | 0 | 0 | 1 | 1 |
| Viola arvensis Murr. | Violaceae | 0 | 0 | 0 | 1 | 1 |
| Viola tricolor L. | Violaceae | 0 | 0 | 0 | 1 | 1 |
| Adenophora lilifolia (L.) A. DC. | Сampanulaceae | 0 | 0 | 0 | 1 | 0 |
| Campanula persicifolia L. | Сampanulaceae | 0 | 0 | 0 | 1 | 0 |
| Campanula trachelium L. | Сampanulaceae | 0 | 0 | 0 | 1 | 0 |
| Campanula glomerata L. | Сampanulaceae | 0 | 0 | 0 | 1 | 0 |
| Campanula patula L. | Сampanulaceae | 0 | 0 | 0 | 1 | 1 |
| Phyteuma spicatum L. | Сampanulaceae | 0 | 0 | 0 | 1 | 0 |

**S5 Table. Summary statistics of elevation, cattle density, fraction of bare soil, soil pH, soil organic carbon content, and density of soil decomposers.** Minimum (min), maximum (max) and mean values, and standard errors ± S.E. are derived across the grasslands for each of the physical-geographical zones and across the entire region; n is the number of grasslands studied here.

| **Study area** | **Summary statistics** | **Elevation,**  m a.s.l. | **Cattle density,**  livestock units h^-1^ | **Bare soil,**  % | **Soil pH** | **Soil organic carbon,**  % | **Soil microorganism abundance,**  cells×10^8^ g^-1^ dry soil | **Biomass of earthworms,**  g m^-2^ |
| --- | --- | --- | --- | --- | --- | --- | --- | --- |
| Across  the entire region (n=31) | min | 137.5 | 0.01 | 0 | 4.1 | 1.10 | 0.001 | 0.01 |
|  | max | 1323.1 | 1.38 | 37.5 | 7.7 | 3.20 | 2.82 | 11.10 |
|  | mean | 399.0 | 0.30 | 5.53 | 5.4 | 1.77 | 0.54 | 3.58 |
|  | ± S.E. | 50.1 | 0.07 | 1.62 | 0.1 | 0.09 | 0.14 | 0.56 |
| Plains  (n=12) | min | 151.3 | 0.02 | 0 | 5.4 | 1.10 | 0.001 | 0.10 |
|  | max | 264.9 | 1.38 | 37.5 | 6.6 | 2.30 | 1.43 | 8.25 |
|  | mean | 218.8 | 0.44 | 9.01 | 5.8 | 1.75 | 0.37 | 2.63 |
|  | ± S.E. | 10.5 | 0.15 | 3.60 | 0.1 | 0.13 | 0.13 | 0.80 |
| Foothills  (n=12) | min | 137.5 | 0.01 | 0.1 | 4.5 | 1.10 | 0.001 | 0.01 |
|  | max | 477.1 | 1.1 | 11.25 | 7.7 | 3.20 | 2.82 | 11.10 |
|  | mean | 323.9 | 0.28 | 2.84 | 5.7 | 2.06 | 0.82 | 5.16 |
|  | ± S.E. | 32.6 | 0.10 | 1.12 | 0.3 | 0.17 | 0.31 | 1.08 |
| Mountains  (n=7) | min | 575.1 | 0.01 | 0.1 | 4.1 | 1.20 | 0.01 | 1.70 |
|  | max | 1323.1 | 0.41 | 19 | 5.1 | 1.41 | 1.08 | 3.60 |
|  | mean | 836.4 | 0.09 | 4.17 | 4.4 | 1.32 | 0.34 | 2.49 |
|  | ± S.E. | 95.6 | 0.05 | 2.75 | 0.1 | 0.03 | 0.16 | 0.25 |

**S6 Table. Results of the final SEM model.** Direct and indirect effects of cattle density and elevation on plant community (χ^2^ = 10.2, df = 9, P = 0.33, RMSEA = 0.07, P_RMSEA_ = 0.39; CFI = 0.99; NNFI = 0.96; SRMR = 0.05). Plant species richness and plant functional diversity were log-transformed; cattle density was square-root transformed. The table shows unstandardized path coefficients (estimate), standard error of regression weight (S.E.), the critical value for regression weight (C.R.), level of significance for regression weight (P), and standardized SEM coefficients (Coeff) for each effect. Significant coefficients (P < 0.05) are given in bold. SOC, soil organic carbon. See also Fig. 3.

| **Path** | | | **estimate** | **S.E.** | **C.R.** | **P** | **Coeff** |
| --- | --- | --- | --- | --- | --- | --- | --- |
| Cattle density | 🡨 | Elevation | <0.001 | <0.001 | -1.95 | **0.05** | **-0.33** |
| Bare soil | 🡨 | Cattle density | 21.37 | 3.52 | 6.07 | **<0.001** | **0.78** |
|  | 🡨 | Elevation | 0.01 | 0.004 | 1.64 | 0.10 | 0.21 |
| Plant species richness | 🡨 | Cattle density | -0.39 | 0.19 | -2.03 | **0.04** | **-0.29** |
|  | 🡨 | Elevation | <0.001 | <0.001 | 2.95 | **0.003** | **0.42** |
|  | 🡨 | Bare soil | -0.01 | 0.01 | -1.40 | 0.16 | -0.19 |
|  | 🡨 | pH | -0.03 | 0.08 | -0.32 | 0.75 | -0.05 |
|  | 🡨 | SOC | -0.09 | 0.12 | -0.79 | 0.43 | -0.11 |
|  | 🡨 | Earthworm biomass | 0.06 | 0.02 | 3.61 | **<0.001** | **0.40** |
|  | 🡨 | Microorganism abundance | 0.14 | 0.07 | 1.83 | 0.07 | 0.23 |
| Plant functional diversity | 🡨 | Plant species richness | 0.14 | 0.15 | 0.96 | 0.34 | 0.18 |
|  | 🡨 | Cattle density | -0.27 | 0.23 | -1.16 | 0.25 | -0.25 |
|  | 🡨 | Bare soil | -0.01 | 0.01 | -2.02 | **0.04** | **-0.37** |
| Proportion of undesirable species | 🡨 | Plant species richness | 1.39 | 6.88 | 0.20 | 0.84 | 0.06 |
|  | 🡨 | Elevation | -0.01 | 0.01 | -1.21 | 0.23 | -0.32 |
|  | 🡨 | Cattle density | -2.54 | 7.78 | -0.33 | 0.74 | -0.08 |
|  | 🡨 | Bare soil | 0.73 | 0.26 | 2.81 | **0.004** | **0.63** |
|  | 🡨 | pH | -3.48 | 2.99 | -1.16 | 0.24 | -0.28 |
|  | 🡨 | SOC | -1.89 | 4.50 | -0.42 | 0.67 | -0.10 |
|  | 🡨 | Earthworm biomass | -0.13 | 0.72 | -0.18 | 0.86 | -0.04 |
|  | 🡨 | Microorganism abundance | 1.34 | 2.98 | 0.45 | 0.65 | 0.10 |
| Earthworm biomass | 🡨 | Cattle density | -1.29 | 2.10 | -0.62 | 0.54 | -0.14 |
|  | 🡨 | Bare soil | -0.09 | 0.08 | -1.14 | 0.25 | -0.25 |
|  | 🡨 | pH | 1.52 | 0.59 | 2.58 | **0.01** | **0.40** |
| Microorganism abundance | 🡨 | Cattle density | -0.61 | 0.45 | -1.36 | 0.17 | -0.26 |
|  | 🡨 | Bare soil | -0.02 | 0.02 | -0.95 | 0.34 | -0.18 |
|  | 🡨 | pH | 0.51 | 0.13 | 4.06 | **<0.001** | **0.55** |
| Soil pH | 🡨 | Elevation | 0.002 | <0.001 | -5.58 | **<0.001** | **-0.75** |
|  | 🡨 | Cattle density | -0.36 | 0.34 | -1.06 | 0.29 | -0.14 |
| SOC | 🡨 | Cattle density | -0.10 | 0.29 | -0.35 | 0.72 | -0.06 |
|  | 🡨 | Elevation | <0.001 | <0.001 | -2.35 | **0.02** | **-0.41** |
|  | 🡨 | Bare soil | -0.01 | 0.01 | -0.55 | 0.58 | -0.10 |
|  | 🡨 | pH | -0.05 | 0.12 | -0.45 | 0.65 | -0.09 |
|  | 🡨 | Earthworm biomass | 0.05 | 0.02 | 2.37 | **0.02** | **0.32** |
|  | 🡨 | Microorganism abundance | 0.25 | 0.11 | 2.36 | **0.02** | **0.36** |

**S7 Table. Selected direct, indirect and total effects of cattle density and elevation on plant species richness, plant functional diversity, and proportion of undesirable species.** The table shows standardized SEM coefficients (Coeff) for each effect and level of significance for regression weight (P). Significant effects (P < 0.05) are given in bold and marginally significant effects (P < 0.09) are given in italic bold. SOC, soil organic carbon.

| **Direct, indirect and total effects of cattle density and elevation** | **P** | **Coeff** |
| --- | --- | --- |
| **Effects of cattle density on plant species richness:** |  |  |
| *Direct effect:* |  |  |
| Cattle density 🡪 Plant species richness | **0.04** | **-0.29** |
| *Indirect Pathways:* |  |  |
| Cattle density 🡪 Bare soil 🡪 Plant species richness | 0.17 | -0.14 |
| Cattle density 🡪 pH 🡪 Plant species richness | 0.76 | 0.01 |
| Cattle density 🡪 Earthworms 🡪 Plant species richness | 0.54 | -0.05 |
| Cattle density 🡪 Microorganisms 🡪 Plant species richness | 0.27 | -0.06 |
| Cattle density 🡪 SOC 🡪 Plant species richness | 0.75 | 0.01 |
| Cattle density 🡪 Bare soil 🡪 Microorganisms 🡪 Plant species richness | 0.18 | 0.04 |
| Cattle density 🡪 Bare soil 🡪 Earthworms 🡪 Plant species richness | 0.10 | -0.10 |
| Cattle density 🡪 Bare soil 🡪 SOC 🡪 Plant species richness | 0.65 | 0.01 |
| Cattle density 🡪 Earthworms 🡪 SOC 🡪 Plant species richness | 0.63 | 0.005 |
| Cattle density 🡪 Microorganisms 🡪 SOC 🡪 Plant species richness | 0.51 | 0.01 |
| Cattle density 🡪 pH 🡪 SOC 🡪 Plant species richness | 0.71 | 0.00 |
| Cattle density 🡪 Bare soil 🡪 Microorganisms 🡪 SOC 🡪 Plant species richness | 0.56 | 0.01 |
| Cattle density 🡪 Bare soil 🡪 Earthworms 🡪 SOC 🡪 Plant species richness | 0.53 | 0.01 |
| *Total Indirect Effect* | 0.72 | -0.28 |
| *Total (direct + indirect)* | **0.049** | **-0.57** |
| **Effects of elevation on plant species richness:** |  |  |
| *Direct effect:* |  |  |
| Elevation 🡪 Plant species richness | **0.003** | **0.42** |
| *Indirect Pathways:* |  |  |
| Elevation 🡪 Cattle density 🡪 Plant species richness | 0.16 | 0.10 |
| Elevation 🡪 Bare soil 🡪 Plant species richness | 0.29 | -0.04 |
| Elevation 🡪 Cattle density 🡪 Bare soil 🡪 Plant species richness | 0.26 | 0.05 |
| Elevation 🡪 SOC 🡪 Plant species richness | 0.46 | 0.04 |
| Elevation 🡪 pH 🡪 SOC 🡪 Plant species richness | 0.70 | -0.01 |
| Elevation 🡪 pH 🡪 Microorganisms 🡪 Plant species richness | 0.11 | -0.10 |
| Elevation 🡪 pH 🡪 Earthworms 🡪 Plant species richness | **0.049** | **-0.12** |
| Elevation 🡪 pH 🡪 Microorganisms 🡪 SOC 🡪 Plant species richness | 0.47 | 0.02 |
| Elevation 🡪 pH 🡪 Earthworms 🡪 SOC 🡪 Plant species richness | 0.48 | 0.01 |
| *Total Indirect Effect* | 0.72 | -0.05 |
| *Total (direct + indirect)* | **0.05** | **0.35** |
| **Effects of cattle density on plant functional diversity:** |  |  |
| *Direct effect:* |  |  |
| Cattle density 🡪 Plant functional diversity | 0.25 | -0.25 |
| *Indirect Pathways:* |  |  |
| Cattle density 🡪 Plant species richness 🡪 Plant functional diversity | 0.39 | -0.05 |
| Cattle density 🡪 Bare soil 🡪 Plant functional diversity | ***0.06*** | ***-0.29*** |
| *Total Indirect Effect* | **0.03** | **-0.34** |
| *Total (direct + indirect)* | **<0.001** | **-0.59** |
| **Effects of elevation on plant functional diversity:** |  |  |
| *Indirect Pathways:* |  |  |
| Elevation 🡪 Plant species richness 🡪 Plant functional diversity | 0.36 | 0.08 |
| Elevation 🡪 Cattle density 🡪 Plant functional diversity | 0.32 | 0.08 |
| Elevation 🡪 Bare soil 🡪 Plant functional diversity | 0.20 | -0.08 |
| Elevation 🡪 Cattle density 🡪 Bare soil 🡪 Plant functional diversity | 0.17 | 0.10 |
| Elevation 🡪 Cattle density 🡪 Plant species richness 🡪 Plant functional diversity | 0.43 | 0.02 |
| Elevation 🡪 Bare soil 🡪 Plant species richness🡪 Plant functional diversity | 0.48 | -0.01 |
| *Total Indirect Effect* | 0.17 | 0.18 |
| **Effects of cattle density on proportion of undesirable species:** |  |  |
| *Direct effect:* |  |  |
| Cattle density 🡪 Proportion of undesirable species | 0.74 | -0.08 |
| *Indirect Pathways:* |  |  |
| Cattle density 🡪 Plant species richness 🡪 Proportion of undesirable species | 0.84 | -0.02 |
| Cattle density 🡪 Bare soil 🡪 Proportion of undesirable species | **0.01** | **0.49** |
| Cattle density 🡪 pH 🡪 Proportion of undesirable species | 0.43 | 0.04 |
| Cattle density 🡪 Earthworms 🡪 Proportion of undesirable species | 0.86 | 0.01 |
| Cattle density 🡪 Microorganisms 🡪 Proportion of undesirable species | 0.67 | -0.03 |
| Cattle density 🡪 SOC 🡪 Proportion of undesirable species | 0.79 | 0.01 |
| Cattle density 🡪 Bare soil 🡪 Plant species richness 🡪 Proportion of undesirable species | 0.84 | -0.01 |
| Cattle density 🡪 Bare soil 🡪 Microorganisms 🡪 Proportion of undesirable species | 0.68 | -0.01 |
| Cattle density 🡪 Bare soil 🡪 Earthworms 🡪 Proportion of undesirable species | 0.86 | 0.01 |
| Cattle density 🡪 Bare soil 🡪 SOC 🡪 Proportion of undesirable species | 0.74 | 0.01 |
| Cattle density 🡪 Earthworms 🡪 SOC 🡪 Proportion of undesirable species | 0.73 | 0.004 |
| Cattle density 🡪 Microorganisms 🡪 SOC 🡪 Proportion of undesirable species | 0.69 | 0.01 |
| Cattle density 🡪 pH 🡪 SOC 🡪 Proportion of undesirable species | 0.77 | -0.001 |
| Cattle density 🡪 Bare soil 🡪 Microorganisms 🡪 SOC 🡪 Proportion of undesirable species | 0.70 | 0.005 |
| Cattle density 🡪 Bare soil 🡪 Earthworms 🡪 SOC 🡪 Proportion of undesirable species | 0.70 | 0.01 |
| *Total Indirect Effect* | **0.01** | **0.52** |
| *Total (direct + indirect)* | **0.02** | **0.44** |
| **Effects of elevation on proportion of undesirable species:** |  |  |
| *Direct effect:* |  |  |
| Elevation 🡪 Proportion of undesirable species | 0.23 | -0.32 |
| *Indirect Pathways:* |  |  |
| Elevation 🡪 Plant species richness 🡪 Proportion of undesirable species | 0.84 | 0.03 |
| Elevation 🡪 Cattle density 🡪 Proportion of undesirable species | 0.30 | 0.11 |
| Elevation 🡪 Bare soil 🡪 Proportion of undesirable species | 0.16 | 0.13 |
| Elevation 🡪 Cattle density 🡪 Bare soil 🡪 Proportion of undesirable species | 0.12 | -0.16 |
| Elevation 🡪 Bare soil 🡪 Plant species richness 🡪 Proportion of undesirable species | 0.84 | -0.002 |
| Elevation 🡪 Cattle density 🡪 Plant species richness 🡪 Proportion of undesirable species | 0.84 | 0.01 |
| Elevation 🡪 SOC 🡪 Proportion of undesirable species | 0.30 | 0.11 |
| Elevation 🡪 pH 🡪 SOC 🡪 Proportion of undesirable species | 0.67 | -0.02 |
| Elevation 🡪 pH 🡪 Microorganisms 🡪 Proportion of undesirable species | 0.66 | -0.04 |
| Elevation 🡪 pH 🡪 Earthworms 🡪 Proportion of undesirable species | 0.86 | 0.01 |
| Elevation 🡪 pH 🡪 Microorganisms 🡪 SOC 🡪 Proportion of undesirable species | 0.32 | 0.04 |
| Elevation 🡪 pH 🡪 Earthworms 🡪 SOC 🡪 Proportion of undesirable species | 0.34 | 0.03 |
| *Total Indirect Effect* | 0.77 | 0.24 |
| *Total (direct + indirect)* | 0.35 | -0.08 |

**S8 Table. Mixed model results for species richness of each plant functional group.** Results from linear mixed models testing the effects of cattle density, elevation, soil properties, and soil biota on species richness of each plant functional group. Plant species richness and plant functional diversity were ln transformed; cattle density was square-root transformed. Significant variables (P < 0.05) are given in bold. ↑ and ↓ show significant increasing and decreasing effects, respectively. An analysis of variance with sequential sum of squares (type I) was applied.

| Explanatory  variable: | Number of species of functional groups: | | | |
| --- | --- | --- | --- | --- |
|  | Legumes | Grasses | Nonlegume Forbs | Rushes and sedges |
| Plant functional diversity | **F_1,21_=6.34;**  **P=0.02 ↑** | **F_1,21_=17.15;**  **P<0.001 ↑** | **F_1,21_=39.97;**  **P<0.001 ↓** | **F_1,21_=31.9;**  **P<0.001 ↑** |
| Plant species richness | **F_1,21_=14.97; P<0.001 ↑** | F_1,21_= 2.83; P=0.11 | **F_1,21_=** **230.7;**  **P<0.001 ↑** | F_1,21_ =0.27;  P=0.61 |
| Elevation | F_1,21_=1.53;  P=0.23 | F_1,21_=0.03;  P=0.87 | F_1,21_=0.12;  P=0.74 | **F_1,21_=8.11;**  **P=0.009 ↑** |
| Cattle density | **F_1,21_=8.00;**  **P=0.01 ↓** | F_1,21_=0.06;  P=0.82 | F_1,21_<0.001;  P=0.99 | F_1,21_=0.17;  P=0.69 |
| Percent bare soil | F_1,21_=0.39;  P=0.54 | F_1,21_=0.10;  P=0.76 | **F_1,21_=6.08;**  **P=0.02 ↑** | **F_1,21_=7.51;**  **P=0.01 ↑** |
| Soil organic carbon | **F_1,21_=5.36;**  **P=0.03 ↑** | F_1,21_=0.02;  P=0.89 | F_1,21_=0.40;  P=0.53 | F_1,21_=1.31;  P=0.26 |
| Soil pH | F_1,21_= 3.82;  P= 0.06 **↑** | F_1,21_= 0.13;  P=0.72 | F_1,21_= 1.26;  P=0.27 | F_1,21_=0.01;  P=0.91 |
| Biomass of earthworms | F_1,21_= 0.41;  P=0.53 | F_1,21_= 0.50;  P=0.49 | F_1,21_= 0.03;  P=0.86 | F_1,21_=0.19;  P=0.67 |
| Microorganism abundance | F_1,21_= 0.18;  P=0.67 | F_1,21_= 2.58;  P=0.12 | F_1,21_= 1.65;  P=0.21 | F_1,21_=0.22;  P=0.64 |

**S9 Table. PERMANOVA results for plant community composition.** Results of a permutational analysis of variance testing the effects of elevation, cattle density, soil properties, and soil biota on plant community composition, which was assessed based on individual species’ cover. Cattle density was square-root transformed. Significant variables (P < 0.05) are given in bold.

| Explanatory  variable: | Df | R^2^ | F | P |
| --- | --- | --- | --- | --- |
| **Elevation** | **1** | **0.06** | **1.96** | **0.005** |
| **Cattle density** | **1** | **0.05** | **1.63** | **0.026** |
| Percent bare soil | 1 | 0.03 | 1.08 | 0.326 |
| Soil organic carbon | 1 | 0.03 | 0.81 | 0.771 |
| Soil pH | 1 | 0.03 | 0.92 | 0.580 |
| Biomass of earthworms | 1 | 0.04 | 1.32 | 0.124 |
| Microorganism abundance | 1 | 0.02 | 0.64 | 0.946 |
| Elevation : Cattle density | 1 | 0.03 | 0.88 | 0.633 |
| Residuals | 22 | 0.70 |  |  |
| Total | 30 |  |  |  |

**S10 Table. Correlations of predictor variables used in the SEM and NMDS analyses**. Given are Pearson correlation coefficients. Stars represent the following levels of significance: *** ≤ 0.001, ** ≤ 0.01, * < 0.05. Statistically significant correlation coefficients are given in bold. SOC, soil organic carbon.

| *Variables* | Cattle density | Bare soil | Soil pH | SOC | Earthworm biomass | Microorganism abundance |
| --- | --- | --- | --- | --- | --- | --- |
| Elevation | -0.331 | -0.047 | **-0.699***** | **-0.440*** | -0.190 | -0.169 |
| Cattle density |  | **0.662***** | 0.107 | -0.211 | -0.284 | -0.359 |
| Bare soil |  |  | 0.021 | **-0.360*** | -0.340 | **-0.360*** |
| Soil pH |  |  |  | **0.498*** | **0.385*** | **0.525*** |
| SOC |  |  |  |  | **0.628***** | **0.631***** |
| Earthworm biomass |  |  |  |  |  | **0.613***** |

**S11 Table. Moran’s I statistics**. Results of the Moran’s I statistic for spatial autocorrelation for residuals of the models for each response variable as tested in SEM (Fig.1, S6 Table), by a linear model (S8 Table), and in PERMANOVA (S9 Table). Moran’s I statistics: *Obs.I* is the computed Moran's I; *Exp.I*, is the expected value of Moran’s I under the null hypothesis (i.e., of no spatial autocorrelation); and *P* stands for the P-value of the test of the null hypothesis against the alternative hypothesis (i.e., of present spatial autocorrelation). Significant variables (P < 0.05) are given in bold. If *Obs.I* is significantly greater than *Exp.I*, then residuals are positively autocorrelated, if *Obs.I* is significantly less than *Exp.I* this indicates negative autocorrelation of residuals.

| *Variable* | *Predictors*  (models for residuals) | ***Moran’s I statistic*** | | |
| --- | --- | --- | --- | --- |
|  |  | *Obs.I* | *Exp.I* | *P* |
| ***Variables as tested in SEM*** (Fig.1, S6 Table)***:*** | | | | |
| Plant species richness | ~ cattle density + elevation + bare soil + pH + SOC + earthworm biomass + microorganism abundance | -0.04 | -0.03 | 0.864 |
| Plant functional diversity | ~ plant species richness + cattle density + bare soil | -0.06 | -0.03 | 0.521 |
| Undesirable species, % | ~ plant species richness + elevation + cattle density + bare soil + pH + SOC + earthworm biomass + microorganism abundance | -0.02 | -0.03 | 0.820 |
| Elevation | — | — | | |
| Cattle density | ~ elevation | -0.03 | -0.03 | 0.887 |
| Bare soil | ~ cattle density + elevation | -0.05 | -0.03 | 0.740 |
| Soil pH | ~ elevation + cattle density | 0.06 | -0.03 | **0.017** |
| SOC | ~ cattle density + elevation + bare soil + pH + earthworm biomass + microorganism abundance | -0.09 | -0.03 | 0.206 |
| Earthworm biomass | ~ cattle density + bare soil + pH | 0.03 | -0.03 | 0.136 |
| Microorganism abundance | ~ cattle density + bare soil + pH | 0.01 | -0.03 | 0.356 |
| ***Number of species of functional groups, as tested by a linear model*** (S8 Table)***:*** | | | | |
| Legumes | ~ plant functional diversity + plant species richness + elevation + cattle density + bare soil + SOC + pH earthworm biomass + microorganism abundance | -0.01 | -0.03 | 0.637 |
| Grasses |  | -0.06 | -0.03 | 0.592 |
| Nonlegume Forbs |  | -0.05 | -0.03 | 0.731 |
| Rushes and sedges |  | -0.04 | -0.03 | 0.811 |
| ***Community composition as tested in PERMANOVA*** (S9 Table)***:*** | | | | |
| NMDS 1 | ~ plant functional diversity + plant species richness + elevation + cattle density + bare soil + SOC + pH earthworm biomass + microorganism abundance + elevation : cattle density | -0.02 | -0.03 | 0.844 |
| NMDS 2 |  | -0.06 | -0.03 | 0.591 |
| NMDS 3 |  | -0.03 | -0.03 | 0.973 |

**Supplementary References**

1. FAO. World reference base for soil resources 2014. International soil classification system for naming soils and creating legends for soil maps. World Soil Resources Reports No. 106. 2014. doi:10.1017/S0014479706394902

2. Polchyna SM, Nikorych VA, Danchu OA. Application of modern FAO/WRB soil classifications system to the Chernivtsy Region map of a soil cover. Gruntoznavstvo (Soil Science). 2004;5: 27–33.

3. QGIS.org (2018). QGIS Geographic Information System. Open Source Geospatial Foundation Project. http://qgis.org.

4. Milchunas DG, Sala OE, Lauenroth WK. A Generalized Model of the Effects of Grazing by Large Herbivores on Grassland. The American Naturalist. 1988;132: 87–106.

5. Whitmore A. Impact of Livestock on Soil. In: Hartung J, Wathes CM, editors. Livestock Farming and the Environment. Hannover; 2001. pp. 39–41.

6. Báldi A, Batáry P, Kleijn D. Effects of grazing and biogeographic regions on grassland biodiversity in Hungary - analysing assemblages of 1200 species. Agriculture, Ecosystems and Environment. Elsevier B.V.; 2013;166: 28–34. doi:10.1016/j.agee.2012.03.005

7. Hart RH, Samuel MJ, Test PS, Smith MA, Hart RH, Samuel MJ, et al. Cattle , Vegetation , and Economic Responses to Grazing Systems and Grazing Pressure. Journal of Range Management. 1988;41: 282–286.

8. Olff H, Ritchie ME. Effects of herbivores on grassland plant diversity. Trends in Ecology and Evolution. 1998;13: 261–265. doi:10.1016/S0169-5347(98)01364-0

9. Grace JB, Jutila H. The Relationship between Species Density and Community Biomass in Grazed and Ungrazed Coastal Meadows. Oikos. 1999;85: 398–408.

10. Warren SD, Thurow TL, Blackburn WH, Garza NE. The Influence of Livestock Trampling under Intensive Rotation Grazing on Soil Hydrologic Characteristics. Journal of Range Management. 1986;39: 491–495. doi:10.2307/3898755

11. Sheath GW, Carlson WT. Impact of cattle treading on hill land: 1. Soil damage patterns and pasture status. New Zealand Journal of Agricultural Research. 1998;41: 271–278. doi:10.1080/00288233.1998.9513311

12. Hobbs RJ. Synergisms among habitat fragmentation, livestock grazing, and biotic invasion in Southwestern Australia. Conservation Biology. 2001;15: 1522–1528.

13. Adler PB, Lauenroth WK. Livestock exclusion increases the spatial heterogeneity of vegetation in Colorado shortgrass steppe. Applied Vegetation Science. 2000;3: 213–222. doi:10.2307/1479000

14. Nevens F, Reheul D. The consequences of wheel-induced soil compaction and subsoiling for silage maize on a sandy loam soil in Belgium. Soil and Tillage Research. 2003;70: 175–184.

15. Grime JP. Competitive exclusion in herbaceous vegetation. Nature. 1973;242: 344–347. doi:10.1038/242344a0

16. Bardgett RD, Wardle DA. Herbivore-mediated linkages between aboveground and belowground communities. Ecology. 2003;84: 2258–2268. doi:10.1890/02-0274

17. Critter SAM, Freitas SS, Airoldi C. Comparison between microorganism counting and a calorimetric method applied to tropical soils. Thermochimica Acta. 2002;394: 133–144.

18. Parham JA, Deng SP, Da HN, Sun HY, Raun W. Long-term cattle manure application in soil. II. Effect on soil microbial populations and community structure. Biology and Fertility of Soils. 2003;38: 209–215. doi:10.1007/s00374-003-0657-7

19. Sun R, Zhang X, Guo X, Wang D, Chu H. Bacterial diversity in soils subjected to long-term chemical fertilization can be more stably maintained with the addition of livestock manure than wheat straw. Soil Biology and Biochemistry. Elsevier Ltd; 2015;88: 9–18. doi:10.1016/j.soilbio.2015.05.007

20. Bertram J. Effects of cow urine and its constituents on soil microbial populations and nitrous oxide emissions. Lincoln University. 2009.

21. Halley BA, VandenHeuvel WJA, Wislocki PG. Environmental effects of the usage of avermectins in livestock. Veterinary Parasitology. 1993;48: 109–125. doi:10.1016/0304-4017(93)90149-H

22. Giller KE, Witter E, Mcgrath SP. Toxicity of heavy metals to microorganisms and microbial processes in agricultural soils: A review. Soil Biology and Biochemistry. 1998;30: 1389–1414. doi:10.1016/S0038-0717(97)00270-8

23. Griffiths BS, Ritz K, Bardgett RD, Cook R, Christensen S, Ekelund F, et al. Ecosystem response of pasture soil communities to fumigation-induced microbial diversity reductions: an examination of the biodiversity–ecosystem function relationship. Oikos. 2000;90: 279–294. doi:10.2307/3547138

24. Breland TA, Hansen S. Nitrogen mineralization and microbial biomass as affected by soil compaction. Soil Biology and Biochemistry. 1996;28: 655–663.

25. Whalley WR, Dumitru E, Dexter AR. Biological effects of soil compaction. Soil and Tillage Research. 1995;35: 53–68. doi:10.1016/0167-1987(95)00473-6

26. Wurst S, Langel R, Scheu S. Do endogeic earthworms change plant competition ? A microcosm study. Plant and Soil. 2005;271: 123–130. doi:10.1007/s11104-004-2201-4

27. Eisenhauer N, Milcu A, Nitschke N, Sabais ACW, Scherber C, Scheu S. Earthworm and belowground competition effects on plant productivity in a plant diversity gradient. Oecologia. 2009;161: 291–301. doi:10.1007/s00442-009-1374-1

28. Kretzschmar A. Burrowing ability of the earthworm Aporrectodea longa limited by soil compaction and water potential. Biology and Fertility of Soils. 1991;11: 48–51. doi:10.1007/BF00335834

29. Buck C, Langmaack M, Schrader S. Influence of mulch and soil compaction on earthworm cast properties. Applied Soil Ecology. 2000;14: 223–229. doi:10.1016/S0929-1393(00)00054-8

30. Silva SR, Silva IR da, Barros NF de, Sá Mendonça E de. Effect of compaction on microbial activity and carbon and nitrogen transformations in two oxisols with different mineralogy. Revista Brasileira de Ciência do Solo. 2011;35: 1141–1149. doi:10.1590/S0100-06832011000400007

31. Van Veen JA, Kuikman PJ. Soil structural aspects of decomposition of organic matter by microorganisms. Biogeochemistry. 1990;11: 213–233. doi:10.1007/BF00004497

32. De Neve S, Hofman G. Influence of soil compaction on carbon and nitrogen mineralization of soil organic matter and crop residues. Biology and Fertility of Soils. 2000;30: 544–549. doi:10.1007/s003740050034

33. Marsh R, Campling R. Fouling of pastures by dung. Herbage Abstracts. 1970;40: 123–130.

34. Holter P. Effect of earthworms on the disappearance rate of cattle droppings. In: Satchell JE, editor. Earthworm Ecology From Darwin to Vermiculture. Springer, Dordrecht; 1983. pp. 49–57.

35. Bagyaraj DJ, Nethravathi CJ, Nitin KS. Soil biodiversity and arthropods: role in soil fertility. In: Chakravarthy AK, Sridhara S, editors. Economic and Ecological Significance of Arthropods in Diversified Ecosystems. Springer Science, Business Media Singapore; 2016. pp. 17–51. doi:10.1007/978-981-10-1524-3

36. Wall DH, Moore JC. Interactions Underground: Soil biodiversity, mutualism, and ecosystem processes. BioScience. 1999;49: 109–117. doi:10.2307/1313536

37. Zonn SV, Travleev AP. Aluminium: Role in Pedogenesis and Influence on the Plants. Dnepropetrovsk: Dnepropetrovsk State Univiversity; 1992.

38. Milchunas DG, Lauenroth WK, Burke IC. Livestock Grazing: Animal and Plant Biodiversity of Shortgrass Steppe and the Relationship to Ecosystem Function. Oikos. 1998;83: 65–74.

39. Williams PH, Hedley MJ, Gregg PEH. Effect of dairy cow urine on potassium adsorption by soil. New Zealand Journal of Agricultural Research. 1988. doi:10.1080/00288233.1988.10423438

40. During C, Weeda WC. Some effects of cattle dung on soil properties, pasture production, and nutrient uptake. New Zealand Journal of Agricultural Research. 1973;16: 423–430. doi:10.1080/00288233.1973.10421125

41. Baldrian P, Šnajdr J. Lignocellulose-degrading enzymes in soils. In: Shukla G, Varma A, editors. Soil Enzymology Soil Biology. Springer, Berlin, Heidelberg; 2010. pp. 167–186.

42. Gough L, Grace JB, Taylor KL, The KL. The Relationship between Species Richness and Community Biomass : The Importance of Environmental Variables. Oikos. 1994;70: 271–279.

43. Mráz P, Ronikier M. Biogeography of the Carpathians: evolutionary and spatial facets of biodiversity. Biological Journal of the Linnean Society. 2016;119: 528–559. doi:10.1111/bij.12918

44. The Statistical Yearbook of the Chernivtsi Region, 2005. Main Department of Statistics in Chernivtsi Region. State Statistics Service of Ukraine. Chernivtsi; 2006.

45. Nikorych V, Szymański W, Skiba M. Redoximorphic Features in Albeluvisols from South-Western Ukraine. Soil Science Working for a Living. 2017. pp. 9–28. doi:10.1007/978-3-319-45417-7

46. Kanivets S. The Factors and Conditions of Soil Formation: A Critical Analysis of Equivalence. Soil Science Working for a Living. 2017. pp. 3–8.

47. Augustine DJ, Booth DT, Cox SE, Derner JD. Grazing intensity and spatial heterogeneity in bare soil in a grazing-resistant grassland. Rangeland Ecology and Management. 2012;65: 39–46. doi:10.2111/REM-D-11-00005.1

48. Eurostat. Statistics Explained [Internet]. 2019 p. https://ec.europa.eu/eurostat/statistics-explained. Available: https://ec.europa.eu/eurostat/statistics-explained/

49. Ramenskij LG, Cacenkin IA, Chizhikov ON, Antipov NA. Ecological assesment of grazing lands based on vegetation [in Russion]. Moscow: Selhosgis; 1956.

50. Medvedev PF, Smetannikova AI. The forage plants of the European part of the USSR [In Russion]. Leningrad: Kolos; 1981.

51. Cacenkin IA. Ecological assesment of grazing lands of the Carpathians and Balkans based on vegetation [in Russion]. Moscow: Institute of Forages; 1870.
