# Supplementary material for: Direct and indirect effects of land-use intensity on plant communities across elevation in semi-natural grasslands
Source: PLoS One. 2020 Nov 24;15(11):e0231122. doi: 10.1371/journal.pone.0231122 (PMC7685434; doi:10.1371/journal.pone.0231122)
Supplement: Attachment — Submitted filename: PONE-D-20-07402-Review.docx [file pone.0231122.s002.docx]

This manuscript presents the results of a study across 31 grassland sites that represent an elevational and cattle density gradient in the eastern Carpathians. In particular, I appreciate that the authors present this complex issue in a way that is detailed and shows that complexity rather than oversimplifying it. There are three major take home messages: 1. Cattle grazing intensity both directly and indirectly decreases plant species richness and functional diversity and increases the proportion of the community that is considered undesirable weeds. 2. Elevation had a strong direct positive effect on plant species richness but also two negative indirect effects. 2. The effect of cattle grazing does not change with increasing elevation. In general, I find these results to be compelling and important and likely address some inconsistencies within the literature that are pointed out by the authors. However, I have two major concerns regarding the analyses presented.

1. Spatial autocorrelation – I could not find in the manuscript a discussion of how spatial autocorrelation was handled. This may have important implications for the results as the plots appear to be collocated in three different regions which are also associated with elevation and cattle density and how close they are to each other may also exert some control over species richness. It could be that I’ve just missed this – in which case I would ask that the authors highlight this a bit more prominently. In the event that I didn’t miss this – I don’t have a particular favorite way of addressing spatial autocorrelation but I’ve included a methods paper that discusses the pros and cons of some different methods (Dormann et al. Ecography). I would be happy with any of these methods as long as spatial autocorrelation is accounted for.
2. The community composition analysis using the NMDS is not well introduced in the introduction and the hypothesis is not as clear to me as the hypothesis for the SEM. I would suggest either removing it or providing a specific hypothesis that is introduced in the introduction along with the predictions for the SEM.

Minor comments:

Undesirable weeds is a tricky thing to say and I would like a description of how species were identified as undesirable. I would also maybe consider different language that isn’t so values oriented, depending on how the species were identified as undesirable maybe non-forage species would be applicable?

24 – land use should be “land use change”

26 – indirect effects should be “the indirect effects”

27 – direct should be “the direct”

34 – direct should be “the direct”

44 – I would say along an elevation gradient here instead

45 – with elevation should be “with increasing elevation”, I think throughout

58 – I would appreciate a few examples of what ecosystem functions and services you are referencing

65 – land use intensity should be “the land use intensity”

67 – I would change can to may here – or could whichever you prefer (I prefer may, ☺ )

70-116 – I really appreciate the complexity here and how thorough you are with the predictions. I would make sure however, that each of these paragraphs has a sentence at the beginning that signals the intention of the paragraph and gives an overarching topic that isn’t linked directly to the first prediction on the list. You do this quite well with the paragraph starting on line 70.

117- 127 – I would consider moving this paragraph up to before you start your prediction paragraphs as it outlines your argument about the novelty and importance of this paper.

292 – I would say “meet the assumptions of normality…” rather than meet

Discussion – I would appreciate a paragraph that goes back to the idea introduced in the introduction about the conflicting results seen in previous studies. Do these results explain some of those conflicts?
